# Postoperative Bio-Chemoradiotherapy Using Cetuximab and Docetaxel in Patients With Cis-Platinum–Intolerant Core High-Risk Head and Neck Cancer: Protocol of a Phase 2 Nonrandomized Clinical Trial

**DOI:** 10.2196/11003

**Published:** 2018-08-23

**Authors:** Goshi Nishimura, Hiromitsu Hatakeyama, Osamu Shiono, Masataka Taguri, Masanori Komatsu, Daisuke Sano, Naoko Sakuma, Kenichiro Yabuki, Yasuhiro Arai, Kunihiko Shibata, Yoshihiro Chiba, Teruhiko Tanabe, Nobuhiko Oridate

**Affiliations:** ^1^ Department of Otorhinolaryngology, Head and Neck Surgery School of Medicine Yokohama City University Yokohama Japan; ^2^ Department of Otorhinolaryngology Yokohama City University Medical Center Yokohama Japan; ^3^ Department of Otorhinolaryngology Yokohama Rosai Hospital Yokohama Japan; ^4^ Department of Data Science School of Data Science Yokohama City University Yokohama Japan

**Keywords:** core high-risk head and neck cancer, postoperative bio-chemoradiotherapy, cetuximab, docetaxel, cis-platinum intolerant

## Abstract

**Background:**

We confirmed the safety of postoperative bio-chemoradiotherapy using cetuximab and docetaxel in a small number of patients with cis-platinum–intolerant core high-risk head and neck cancer.

**Objective:**

To assess treatment efficacy, we planned a phase 2 study of postoperative bio-chemoradiotherapy for patients with cis-platinum–intolerant core high-risk head and neck cancer and will compare the results to those of previously collected radiotherapy data.

**Methods:**

Patients who underwent definitive surgery for oral cavity, laryngeal, oropharyngeal, or hypopharyngeal advanced cancer, whose postoperative pathological results indicated core high risk for recurrence (eg, positive margin in the primary site or extranodal extension) and who were cis-platinum–intolerant, will undergo postoperative bio-chemoradiotherapy. The primary end point is 2-year disease-free survival.

**Results:**

The expected 2-year disease-free survival is set at 55%, and the calculated sample size is 35 patients, according to a statistical analysis based on previous reports.

**Conclusions:**

This treatment method is expected to improve the survival rate of patients with severe head and neck cancer.

**Trial Registration:**

UMIN Clinical Trials Registry UMIN000031835; https://upload.umin.ac.jp/cgi-open-bin/ctr_e/ ctr_view.cgi?recptno=R000036355 (Archived by WebCite at http://www.webcitation.org/71fejVjMr)

## Introduction

Most patients with head and neck cancers present with advanced disease stages during their first hospital visit. For patients with advanced head and neck cancers, surgery is a common definitive therapy. After surgery, a pathological evaluation is necessary to decide upon additional therapy. The pathological risk factors for recurrence and/or distant metastases after surgery are as follows: positive surgical margins, T3 and T4 pathologies, positive perineural invasions and/or vascular tumor embolism of the primary site, and extranodal extension (ENE) and/or multiple positive metastasis to lymph nodes. Patients with these results are recommended to undergo postoperative radiotherapy [1,2]. Among these risk factors, microscopically involved surgical margins of the primary site and ENE are considered to be core high-risk factors that indicate a poor prognosis. The outcome for patients with these core high-risk factors could be improved by concurrent chemotherapy during postoperative radiotherapy [3].

**Figure 1:**
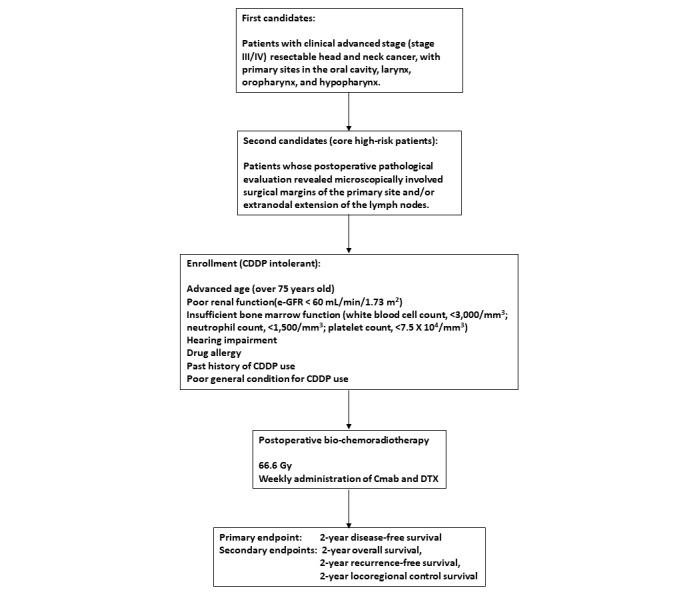
Patient enrollment, treatment, and analysis. CDDP: cis-platinum; Cmab: cetuximab; DTX: docetaxel; e-GFR: estimated glomerular filtration rate.

The standard protocol for postoperative chemoradiotherapy is the concurrent use of cis-platinum (CDDP; 100 mg/m^2^ once every 3 weeks) during radiotherapy (total dose of 66 Gy) for patients with core high-risk factors [1-3]. However, many of these patients are intolerant to CDDP due to advanced age, poor renal function, hearing impairment, and/or poor general condition. One option to overcome these problems is the combined administration of cetuximab (Cmab) and docetaxel (DTX) during postoperative radiotherapy. This regimen has been shown to afford favorable outcomes with improved disease-free survival (DFS) and overall survival (OS) and less toxicity compared to high-dose CDDP administration [4]. A phase 2/3 trial (RTOG 1216) of this regimen is ongoing. Focusing on the reduced toxicity of this regimen, we administered the combination of Cmab and DTX during postoperative radiotherapy for a limited number of patients with CDDP-intolerant core high-risk head and neck cancer, and established the safety of this procedure [5]. Here, we propose a multicenter, single-arm phase 2 trial to confirm the efficacy of postoperative bio-chemoradiotherapy (B-CRT) using Cmab and DTX for patients with CDDP-intolerant core high-risk head and neck cancer (Figure 1).

## Methods

### Study Setting

This will be a multicenter single-arm open-label nonrandomized trial. The central hospital is Yokohama City University Hospital, and the corporate hospitals are Yokohama City University Center Hospital and Yokohama Rosai Hospital.

### End Points

The primary end point is 2-year DFS, and the events are uncontrollability of existing cancer (locoregional remnant, locoregional recurrence, and/or distant metastasis), appearance of new primary cancer, and death. The secondary end points are 2-year OS, 2-year recurrence-free survival (RFS), and 2-year locoregional control survival (LCS). The event of OS is death, events of RFS are locoregional recurrence and distant metastasis of existing cancer and death, and events of LCS are locoregional recurrence and death. In patients with locoregional recurrences of advanced head and neck cancer, 60% to 70% of these recurrences become apparent within 1 year after the initial treatment and 90% to 100% become apparent within 2 years [6,7]. Accordingly, the end point is set at 2 years.

### Ethics Approval

Ethics approval for this study was obtained from the Yokohama City University Institutional Review Board (#B180301010). Written informed consent was obtained from the participants in this study.

### Eligibility Criteria

#### Candidates

Head and neck cancer is classified according to the 8th edition of the TNM Staging System [8]. Patients with clinical advanced stage (stage III/IV) oral cavity, laryngeal, oropharyngeal, and hypopharyngeal carcinomas, which are considered resectable by definitive surgery, are the first candidates. Among the first candidates, patients with a postoperative pathological evaluation that reveals a microscopically involved surgical margin of the primary site or ENE are the second candidates. Among the second candidates, patients defined as CDDP-intolerant are enrolled.

#### Inclusion Criteria

Prior to enrollment in this trial, the patients must meet all of the following criteria: pathologically proven carcinoma; primary tumor located in the oral cavity, larynx, oropharynx, or hypopharynx; clinically advanced stage (stage III or IV) on visual, endoscopic examinations, imaging examinations (eg, computed tomography [CT], magnetic resonance imaging [MRI], ultrasonic echo [US], and positron-emission tomography [PET]-CT); primary site assessed as resectable by definitive surgery and regional lymph node assessment by neck dissection on CT, MRI, or US; no distant metastasis on PET-CT (cM0); age over 20 years (regarded as a legal adult in Japan); performance score (PS) 0 to 2 on Eastern Cooperative Oncology Group (ECOG) criteria; sufficient general condition for operation under general anesthesia; CDDP-intolerant (eg, advanced age [over 75 years], poor renal function [estimated glomerular filtration rate <60 mL/min/1.73 m^2^], insufficient bone marrow function [white blood cell count <3000/mm^3^, neutrophil count <1500/mm^3^, platelet count <7.5×104/mm^3^], hearing impairment, drug allergy, past history of CDDP use, or poor general condition); and provision of written informed consent.

#### Exclusion Criteria

Prior to enrollment in this trial, the patients must not meet any of the following criteria: incurable synchronous malignancies, priority systemic diseases, or refusal to undergo definitive surgery and/or postoperative radiotherapy.

### Treatment Methods

#### Surgery

Definitive primary resection is performed for the primary site with simultaneous neck dissection for node-positive cases.

#### Bio-Chemoradiotherapy

Radiotherapy is administered in conventional fractions of 1.8 Gy for a total dose of 66.6 Gy, 5 days per week, using 4 to 6 megavolt x-rays. The reason for 1.8 Gy dose fraction, not 2.0 Gy, is to reduce radiation-associated adverse events (eg, mucositis and dermatitis), with concurrent use of chemotherapy in the whole-neck field with advanced stage patients. The radiation fields are set up for the primary tumor and prophylactically, the bilateral cervical lymph node area (levels I-V and the retropharyngeal lymph node area). The cervical lymph node area is administered prophylactic doses of 45 Gy, with lateral opposed fields to the upper and anterior lower neck. The B-CRT regimen consists of weekly Cmab (week 1: 400 mg/m^2^; subsequent weeks: 250 mg/m^2^) and weekly DTX (15 mg/m^2^). Cmab is discontinued for grade 3-4 hypersensitivity, and DTX is discontinued for grade 4 hyperinsensitivity. Termination or suspension of Cmab administration, DTX administration, or radiotherapy is considered based on grade 3-4 adverse events or patient status.

#### Adverse Event Evaluation

Adverse events are scored according to the National Cancer Institute’s Common Terminology Criteria for Adverse Events, version 4.0. Patients are assessed once or twice per week during radiotherapy for general status, weight, blood counts, serum levels, and adverse events.

### Scheduled Analysis

The final analysis is scheduled at the end of the observation period. The primary end point is 2-year DFS, and the secondary end points are 2-year OS, 2-year RFS, and 2-year LCS.

### Statistical Analysis

The DSF of patients with postoperative advanced stage (stage III/IV) head and neck cancer who were treated with radiotherapy alone after definitive surgery was 36% at both 3 years and 5 years [1-3]. Postoperative chemoradiotherapy with high-dose CDDP for patients with advanced stage cancer revealed a 23% reduction in treatment failure risks associated with DFS compared with radiotherapy alone. The OS of patients with core high-risk factors (eg, microscopically involved surgical margin of the primary site or ENE) significantly improved through the concurrent use of high-dose CDDP during postoperative radiotherapy [1-3]. The combined use of Cmab and DTX for patients with core high-risk factors during postoperative radiotherapy increased the 2-year DFS by 11% compared with patients who received high-dose CDDP [4]. In this study, considering that CDDP intolerance refers to the poor condition of patients with core high-risk factors, the expected 2-year DFS is set at 35% for patients treated with postoperative radiotherapy alone; Cmab and DTX could increase the survival rate by 20%. Thus, we set the expected 2-year DFS as 55%. Under the 2-tailed significance level of .05 and power of 80%, the required sample size was calculated as 35 for this analysis.

## Results

**Figure 2:**
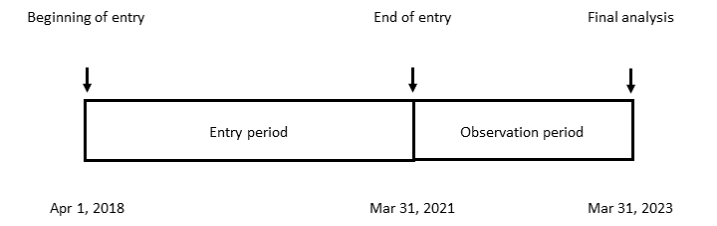
Expected trial schedule.

The University Hospital Medical Information Network Clinical Trials Registry (UMIN000031835) was completed on March 22, 2018. Patient enrollment started on April 1, 2018, and enrollment will close on March 31, 2021. The observation period will end on March 31, 2023. The expected schedule is shown in Figure 2.

## Discussion

Head and neck cancers account for approximately 5% of all malignancies in Japan, and both the incidences and mortality rates are increasing, especially in patients aged over 50 years [9,10]. The risk factors of head and neck cancer are smoking and alcohol consumption, and older patients have a longer history of these habits. Aging and a long history of smoking or alcohol consumption can induce cardiovascular disorders, pulmonary diseases, hepatic disorders, synchronous malignancies, and poor general condition. Additionally, Japanese patients with head and neck cancer show poorer renal functions with age [11], which limits the use of platinum-based drugs, especially CDDP. Such disadvantages restrict ideal treatment and are risk factors for complications during treatment.

Approximately 60% of patients with head and neck cancer present with advanced stage disease at their first visit, and surgery has been the definitive therapy for patients with advanced stage head and neck cancer despite recent improvements in treatment and diagnostic instruments. Although surgery is a fundamental treatment for locoregional control, multidisciplinary approaches, including radiotherapy and chemotherapy, are improving prognoses. Postoperative radiotherapy has been the standard adjuvant therapy for patients with high risks of locoregional recurrences or distant metastases [12,13], but radiotherapy does not show dramatic prognostic improvements. After the report of Bernier et al [3], the concurrent use of high-dose CDDP with postoperative radiotherapy has become the standard therapy for patients with core high-risk factors (eg, microscopically involved surgical margins of the primary site and ENE). Although the accepted standard regimen is CDDP administration at the dose of 100 mg/m^2^ once every 3 weeks during radiotherapy, many patients with core high-risk head and neck cancer are not suited to receive this regimen due to advanced age, poor renal function, hearing impairment, or poor general status.

Cmab is an immunoglobulin G subclass 1 monoclonal antibody that inhibits ligand binding to the epidermal growth factor receptor (EGFR) and stimulates antibody-dependent cell-mediated cytotoxicity. One of the mechanisms of Cmab is that EGFR inhibition will result in decreased proliferation via mitogen-activated protein kinase and phosphatidylinositol-3-kinase pathways [14]. It has been shown that Cmab has synergistic effects with radiotherapy by modification of the radiation response through EGFR and its signal transduction pathways [15-17]. DTX is a taxane drug with a cytotoxic effect that is attributed to the inhibition of microtubule stabilization during cell divisions [18]. DTX has radiosensitizing effects by increasing G2/M phase cell fraction, reoxygenation of radioresistant hypoxic cells, radiation-induced apoptosis, and other synergistic antiangiogenetic effects [19-21]. Cmab and DTX have synergistic chemotherapeutic and radiosensitizing effects both in vivo and in vitro [22].

In a randomized phase 2 trial, Harari et al [4] reported superior 2-year OS and DFS with less toxicity in patients with core high-risk factors who received postoperative radiotherapy with Cmab and DTX compared with those who received high-dose CDDP. The 2-year OS was 79% for the Cmab plus DTX arm and 69% for the CDDP arm, and the 2-year DFS was 66% and 57%, respectively. Grade 3/4 myelosuppression was observed in 14% of patients in the Cmab plus DTX arm and 28% of patients in the CDDP arm. Regarding hematological adverse events, a small number of grade 3/4 events (3% of lymphopenia and 0.5% of anemia [23,24]) were reported with radiotherapy with Cmab alone, and an 8% rate of leukopenia, 4% to 4.8% rate of neutropenia, 56% rate of lymphopenia, and 4.8% rate of thrombocytopenia [25,26] were reported with radiotherapy with DTX alone in patients with advanced head and neck cancer. Although the combined use of Cmab and DTX with radiotherapy tends to result in a high incidence of severe myelosuppression, in our experience it was a low percentage and was controllable compared to the myelosuppression events in patients who were administered CDDP. Following this report, we applied this regimen as postoperative B-CRT in a small number of patients (11) with CDDP-intolerant core high-risk head and neck cancer and confirmed the efficacy and safety [5]. The 2-year DFS was 55%, which was the same as the expected 2-year DFS that was calculated from previous reports in this trial. No grade 4 adverse events were observed, and the grade 3 adverse events included oral mucositis (45%), aspiration (27%), radiation dermatitis (18%), leucopenia (9%), neutropenia (9%), lung infection (9%), and hyponatremia (9%). These adverse events were controllable and tolerable. Both Cmab and DTX are considered to have less usage restrictions compared with CDDP. We consider that these merits make the combined use of Cmab and DTX as postoperative B-CRT suitable for patients with CDDP-intolerant core high-risk head and neck cancer. We will clarify the efficacy of this regimen in this phase 2 trial. A limitation to this study is that it has a single arm with a limited number of patients.

## Abbreviations

B-CRT: bio-chemoradiotherapy
CDDP: cis-platinum
Cmab: cetuximab
CT: computed tomography
DFS: disease-free survival
DTX: docetaxel
ECOG: Eastern Cooperative Oncology Group
EGFR: epidermal growth factor receptor
ENE: extranodal extension
LCS: locoregional control survival
MRI: magnetic resonance imaging
OS: overall survival
PET: positron-emission tomography
RFS: recurrence-free survival
US: ultrasonic echo
